# 

**Published:** 1918-05-18

**Authors:** 


Rates of Subscription: Twelve months, 6/6; Six months, 4/-; three months, 2/-, post free to any address in the United Kingdom. Abroad, Twelve months, 8/8; Six months, 5/-; Three months, 2/6, post free. THE HOSPITAL "Index" to Volume LXIII. October 1917 to March 1918, is now ready, price 3½d. per copy, post free. Address The Manager, The Hospital, "The Hospital" Building, 28/29 Southampton Street, Strand, London, W.C. 2.

### Passing Events and Topics.

### Health Visitors.

The London County Council has made representations to the Local Government Board with a view to the amendment of the Health Visitors (London) Order, 1909, so as to secure that only persons suited by experience and training for the positions shall be appointed in that capacity.

### Musical Instruments for Fighters.

Madame Clara Novello Davies is the organiser of a movement to provide musical instruments for those who can play amongst our fighters upon sea and land. Any kind of instrument will be gladly received at 8-9 Denman Street, Piccadilly.

### The Royal Sanitary Institute.

The Henry Saxon Snell Prize in 1918 of fifty guineas and the medal of the Institute is offered for an essay on "Suggestion for Improvements in Apparatus and Appliances for Dealing with House Refuse."

### War Hospital Activities.

"Subscriptions are being received" is the latest encouraging report from the authorities of the Holborn Division of the Red Cross Society, who are busy at their headquarters in Russell Square making every endeavour towards the realisation of the scheme for a Holborn War Hospital. At a meeting held recently under the presidency of the Mayor it was agreed that such an institution for presentation to the War Office was desirable, and that the sum of £4,000 should be the figure to be aimed at. An effort to raise a similar amount for the continuance of the Weir Hospital, Balham, for another year is being made by the Kensington Division. In addition to a lecture and a concert to be held in aid of the fund, students of the local public and private schools are throwing themselves into the work. Princess Louise is giving her patronage to these endeavours.

### National Baby Week.

The Executive Committee propose that Baby Week celebrations shall be held this year from July 1 to 7; that there shall be a two days' conference in London to discuss infant-welfare matters generally, and in the provinces on dates convenient to each district. A central exhibition for teachers and welfare workers is recommended.

### The College of Ambulance.

A summer course of lectures and demonstrations in ambulance work and first-aid will be held at the College of Ambulance, 3 Vere Street, Cavendish Square, on Thursday afternoons in May, June, and July at 4.30 p.m. An attractive list of lectures has been published.

